# Sarcomatoid malignant pleural mesothelioma: a case of long-term recurrence-free survival following curative intent surgery alone

**DOI:** 10.1186/s40792-024-01939-1

**Published:** 2024-05-31

**Authors:** Masatoshi Kanayama, Masaru Takenaka, Katsuma Yoshimatsu, Hiroki Matsumiya, Masataka Mori, Koji Kuroda, Aya Nawata, Manabu Yasuda, Fumihiro Tanaka

**Affiliations:** 1 Second Department of Surgery, University of Occupational and Environmental Health, Japan, Kitakyushu, Japan; 2 Department of Chest Surgery, Iizuka Hospital, Iizuka, Japan; 3 Department of Pathology and Oncology, School of Medicine, University of Occupational and Environmental Health, Japan, Kitakyushu, Japan

**Keywords:** Surgery, Malignant pleural mesothelioma, Sarcomatoid subtype

## Abstract

**Background:**

Curative intent surgery may be indicated for some patients with resectable early stage malignant pleural mesothelioma (MPM). However, sarcomatoid MPM is a highly aggressive subtype for which curative intent surgery is generally not recommended.

**Case presentation:**

We present the case of a 63-year-old man who presented with dyspnea and chest tightness. Computed tomography revealed pleural thickening and nodular lesions. A pleural biopsy confirmed lymphohistiocytoid MPM (cT1N0M0, stage IA), prompting surgical intervention. The patient underwent left extrapleural pneumonectomy (EPP), and the final diagnosis was sarcomatoid MPM (pT2N0M0, stage IB). Although post-operative chemotherapy was planned, the patient refused additional treatment, because of the introduction of home oxygen therapy, and has remained recurrence-free for 10 years after the surgery.

**Conclusions:**

This case presents a noteworthy instance of achieving long-term recurrence-free survival solely through curative intent surgery for sarcomatoid MPM. It highlights the potential efficacy of surgical intervention in managing this aggressive subtype, offering a glimmer of hope for improved outcomes. Further research is warranted to better define the role of surgery in the treatment of sarcomatoid MPM.

## Background

Curative intent surgery may be indicated for some patients with resectable early stage malignant pleural mesothelioma (MPM). However, sarcomatoid MPM is a highly aggressive subtype for which curative intent surgery is generally not recommended [1]. Here, we present the case of a patient with sarcomatoid MPM who achieved long-term recurrence-free survival solely through curative intent surgery. This case underscores the potential efficacy of curative intent surgery for this challenging subtype.

## Case presentation

**Fig. 1:**
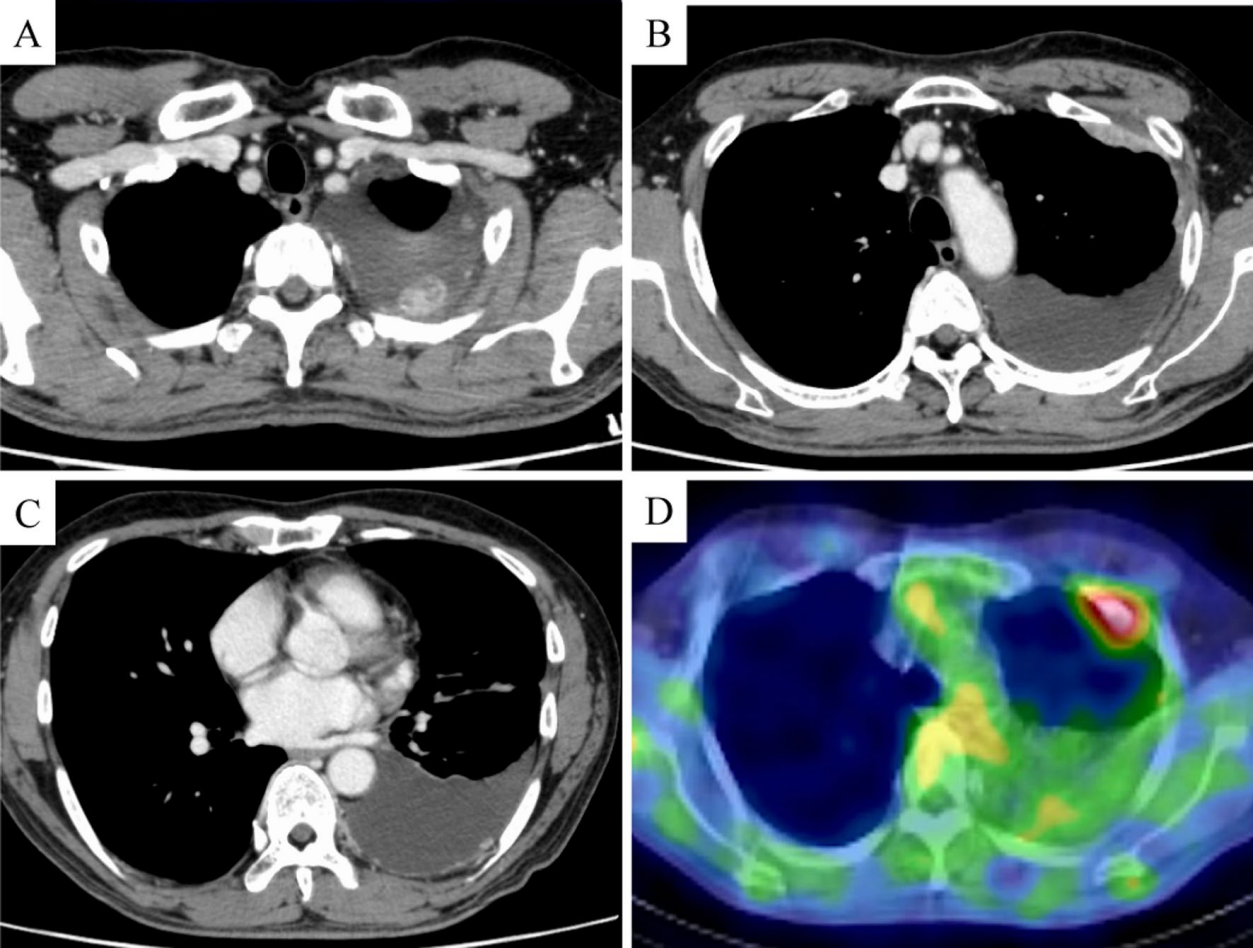
**A**–**C** Contrast-enhanced CT of the chest reveals pleural thickening and pleural nodular lesions with left pleural effusion. **D** PET–CT demonstrates markedly increased FDG uptake within the pleural nodular lesion. *CT* computed tomography, *PET–CT* positron emission tomography–computed tomography, *FDG* fluorodeoxyglucose

**Fig. 2:**
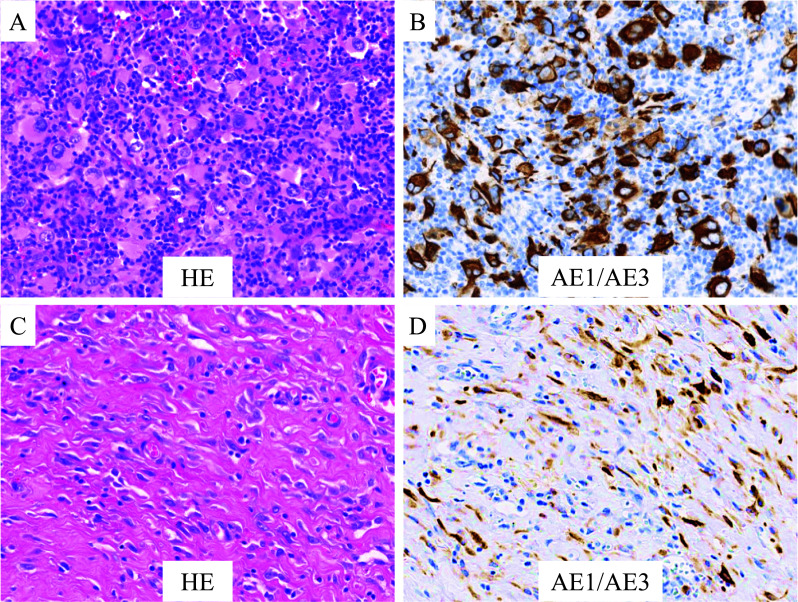
**A** Pleural biopsy specimen shows the proliferation of highly atypical cells with polychromatic pleomorphic nuclei and abundant acidophilic cytoplasm, accompanied by marked chronic inflammation (hematoxylin and eosin staining; original magnification ×100). **B** Atypical cells are diffusely positive for AE1/AE3 (AE1/AE3; original magnification ×100). **C** Surgical specimen reveals diffuse proliferation of atypical spindle cells arranged in a fascicular or haphazard pattern with mitotic figures (hematoxylin and eosin staining; original magnification ×100). **D** Atypical spindle cells are diffusely positive for AE1/AE3 (AE1/AE3; original magnification ×100)

A 63-year-old man was referred to our hospital with dyspnea and left-sided chest tightness. Contrast-enhanced computed tomography of the chest revealed pleural thickening and nodular lesions with left pleural effusion (Fig. 1). Blood test, including tumor markers such as soluble mesothelin-related peptides (SMRP), showed no abnormalities; however, a high level of hyaluronic acid (121,000 ng/mL) in pleural fluid on thoracentesis and a history of asbestos exposure raised the suspicion of MPM. Pleural biopsy revealed lymphohistiocytoide MPM (cT1N0M0 stage IA, Psum: 24 mm; Fig. 2A, B) (Union for International Cancer Control, 8th and 9th ed.), which led us to perform surgery.

The patient underwent left extrapleural pneumonectomy (EPP) with resection and reconstruction of the pericardium and diaphragm. The surgery lasted for 8 h 30 min, with a blood loss of 190 mL. The postoperative course was uneventful, and the thoracic drain was removed on postoperative day 4. The patient was discharged on postoperative day 19. The final diagnosis was sarcomatoid MPM (pT2N0M0, stage IB; Fig. 2C, D) (Union for International Cancer Control, 8th and 9th ed.). Preoperative lymphohistiocytoid MPM was barely detectable in the excised specimens. Although postoperative chemotherapy was planned, the patient refused additional treatment owing to the introduction of home oxygen therapy following EPP and has remained under observation without treatment. Ten years have passed since the surgery, and the patient has remained recurrence-free.

## Conclusions and discussion

This case marks a significant milestone as the first documented instance of long-term recurrence-free survival achieved solely through curative intent surgery for sarcomatoid MPM. It highlights the potential efficacy of surgical intervention in this subtype.

Sarcomatoid MPM is generally associated with a very poor prognosis, and surgical intervention is typically discouraged owing to its limited impact on patient outcomes [1]. Our institution’s historical outcomes for surgical treatment of sarcomatoid MPM have been unfavorable [2], leading us to primarily rely on systemic therapy for this aggressive subtype. However, in this case, initial biopsy results—potentially influenced by tumor heterogeneity—revealed lymphohistiocytoid MPM, which shares characteristics with the epithelioid subtype [3]. This histological finding guided our decision to proceed with curative intent surgery following a comprehensive review by our Cancer Board.

Achieving a curative microscopic resection in MPM is inherently challenging due to its diffuse growth pattern and lack of definitive surgical margins. Typically, multimodal therapy, including chemotherapy and radiotherapy, is required to eradicate residual microscopic disease [4]. Although postoperative multimodal therapy was planned in this case, it was not administered owing to the patient’s need for home oxygen therapy following EPP and personal treatment preferences. At the time of this patient’s treatment, our standard surgical procedure was EPP. However, considering the subsequent challenges in administering multimodal therapy after lung resection, our current approach now favors P/D. While EPP may technically reduce the risk of local recurrence, as indicated by retrospectively achieving R0 resection in this instance, P/D is generally preferred to facilitate adjuvant therapies and may ultimately lead to a lower risk of local recurrence [2].

Large-scale studies utilizing data from the National Cancer Database have demonstrated the efficacy of curative surgery in early stage (cT1-2N0M0) sarcomatoid MPM [5]. Reflecting on this case, curative intent surgery could potentially be beneficial for selected sarcomatoid MPM. In addition, the advent of immunotherapy has opened new avenues for incorporating surgery into multimodal treatment strategies. Emerging evidence indicates that combining surgery with immunotherapy could improve outcomes for patients with poor prognosis sarcomatoid MPM, which should be explored in future clinical trials [6, 7].

In conclusion, this case presents a noteworthy instance of achieving long-term recurrence-free survival solely through curative intent surgery for sarcomatoid MPM. It highlights the potential efficacy of surgical intervention in managing this aggressive subtype, offering a glimmer of hope for improved outcomes. Further research is warranted to better define the role of surgery, potentially in combination with multimodal therapies, in the treatment of sarcomatoid MPM.

## Data Availability

Not applicable.

## Abbreviations

MPM: Malignant pleural mesothelioma
SMRP: Soluble mesothelin-related peptides
EPP: Extrapleural pneumonectomy
P/D: Pleurectomy decortication
